# Trans-species activity of a nonself recognition domain

**DOI:** 10.1186/1471-2180-13-63

**Published:** 2013-03-22

**Authors:** Robert Phillip Smith, Kenji Wellman, Myron L Smith

**Affiliations:** 1 Department of Biology, Carleton University, Ottawa, ON K1S 5B6, Canada; 2 Division of Math, Science and Technology, Nova Southeastern University, Fort Lauderdale, FL 33314, USA

**Keywords:** Nonself recognition, Heterokaryon incompatibility, Ribonucleotide reductase, Heat shock protein, Sexual incompatibility.

## Abstract

**Background:**

The ability to distinguish nonself from self is a fundamental characteristic of biological systems. In the filamentous fungus *Neurospora crassa*, multiple incompatibility genes mediate nonself recognition during vegetative growth. One of these genes, *un*-*24*, encodes both nonself recognition function and the large subunit of a type I ribonucleotide reductase, an evolutionarily conserved enzyme that is essential for the conversion of NDP precursors into dNDPs for use in DNA synthesis. Previous studies have shown that co-expression of the two allelic forms of *un*-*24*, Oakridge (OR) and Panama (PA), in the same cell results in cell death.

**Results:**

We identify a 135 amino acid nonself recognition domain in the C-terminus region of UN-24 that confers an incompatibility-like phenotype when expressed in the yeast, *Saccharomyces cerevisiae*. Low-level expression of this domain results in several cytological and phenotypic characteristics consistent with an incompatibility reaction in filamentous fungi. These incompatibility phenotypes are correlated with the presence of a non-reducible complex consisting of the PA incompatibility domain and Rnr1p, a large subunit of ribonucleotide reductase in yeast. When the PA incompatibility domain is switched to high-level expression, the incompatibility phenotype transitions to wild-type concomitant with the appearance of a complex containing the PA incompatibility domain and Ssa1p, an Hsp70 homolog.

**Conclusions:**

Results from this study provide insights into the mechanism and control of vegetative nonself recognition mediated by ribonucleotide reductase in *N*. *crassa*, thus establishing the yeast system as a powerful tool to study fungal nonself recognition. Our work shows that heat shock proteins may function to deactivate vegetative incompatibility systems, as required for entry into the sexual cycle. Finally, our results suggest that variations on the PA incompatibility domain may serve as novel and specific antimicrobial peptides.

## Background

Nonself recognition systems are ubiquitous in diverse organisms as exemplified by restriction endonucleases in bacteria [1] and the major histocompatibility complex in vertebrates [2]. In filamentous fungi, such as *Neurospora crassa*, nonself recognition occurs in both the sexual and vegetative phases [3]. In the sexual phase, nonself recognition is associated with the mating-type locus and facilitates outbreeding [4]. During the vegetative phase, nonself recognition may occur after cells fuse to form heterokaryotic cells, which contain two or more genetically distinct nuclei [3,5]. In *N*. *crassa*, viability of heterokaryons is governed by heterokaryon incompatibility (*het*) loci [3] where an allelic difference at one or more of these loci results in programmed cell death [5].

As in other filamentous ascomycetes, *N*. *crassa* has multiple *het* loci. One of these, the *un*-*24* gene, has an interesting dual function. In addition to heterokaryon incompatibility, *un*-*24* also encodes the large subunit of a class I ribonucleotide reductase (RNR). Class I RNRs are highly conserved across eukaryotes and operate as tetramers composed of two large subunits and two small subunits that catalyze the reduction of ribonucleoside diphosphates (NDPs) into deoxyribonucleoside diphosphates (dNDPs). The dNDPs are, in turn, phosphorylated to obtain the dNTPs that are essential for *de novo* synthesis of DNA [6-9]. This dual function of *un*-*24* is of particular interest since it implicates a potential connection between DNA synthesis and nonself recognition-associated cell death.

There have been no reports of nonself recognition function by RNRs in organisms outside of *Neurospora*, suggesting that this is a derived characteristic of the *un*-*24* gene. Overall, the predicted UN-24 protein is very similar to other eukaryotic RNR class I large subunits except for a well defined, variable region near the C-terminus [10]. Interestingly, the carboxy termini of the two allelic forms of UN-24 in *N*. *crassa*, Oakridge (OR) and Panama (PA), are strikingly different and bear signatures of diversifying selection [11]. This led us to test whether incompatibility function of UN-24 proteins reside in the C-terminus region, and indeed this is the case; the C-termini of both allelic forms can autonomously trigger an incompatibility reaction when expressed in cells having the opposite allele. We then sought to determine if the UN-24 C-termini from *N*. *crassa* retained activity when expressed in the unicellular yeast *Saccharomyces cerevisiae*. Surprisingly, the 135 amino acid PA incompatibility domain (PAp) is also toxic when expressed in yeast. Given that yeast appears to lack a vegetative nonself recognition system [12], this trans-species incompatibility activity provided an opportunity to explore the mechanism of this nonself recognition domain without interference from other incompatibility factors normally present in *N*. *crassa*.

## Results

### Incompatibility activity and specificity of the UN-24 C-terminus

**Figure 1:**
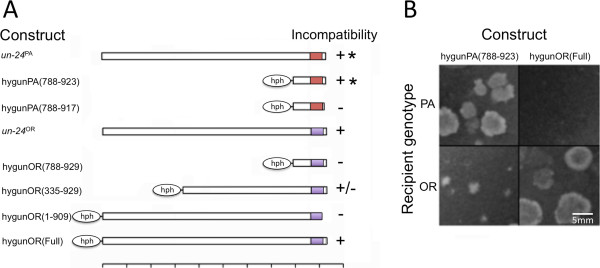
**Incompatibility activity is determined by the C**-**terminus of UN**-**24. A**) Regions of *un*-*24*^PA^ and *un*-*24*^OR^ were fused to the hygromycin resistance gene (*hph*) and tested for incompatibility activity by transformations of PA [C2(2)-1] and OR (C9-2) strains. The red (PA) or purple (OR) region at the right represents the highly variable C-terminus region. At the right of each construct, “+*” indicates PA-like activity, “–” represents no incompatibility activity, “+” designates strong OR-like activity, and “+/−” indicates weak OR-like activity. Each interval on the bottom scale bar represents a length of 100 amino acid residues. **B**) Representative transformation assays of incompatible and compatible interactions in *N*. *crassa*. Transformation of *un*-*24*^PA^ constructs into the OR (C9-2) strain resulted in ‘star’ colonies that are characteristic of PA-like incompatibility. In contrast, transformation of *un*-*24*^OR^ into the PA [C2(2)-1] strain results in near complete cell death of the recipient strain and recovery of few or no transformants, indicative of strong OR-like incompatibility.

The OR and PA UN-24 proteins exhibit significant differences in their ~120 amino acid (aa) C-termini [11] whereas the ~810 aa N-terminal regions are identical. We hypothesized that incompatibility specificity would reside in the C-terminal portion of *un*-*24*. We tested this using constructs consisting of a hygromycin B resistance gene, *hph*, fused in-frame to various fragments of *un*-*24*^PA^ or *un*-*24*^OR^ (Figure 1A). We could infer expression of the fused *un*-*24* domains by virtue of hygromycin B resistance of the transformants. Incompatibility activity of these constructs was tested by transforming them into C9-2 (*un*-*24*^OR^) and C2(2)-1 (*un*-*24*^PA^) strains and examining transformant viability and/or phenotype (Figure 1B). In our naming scheme the range of UN-24 amino acid residues included in the fusion gene product is given in parentheses. For example, the hygunPA(788–923) construct that contained the *un*-*24*^PA^ region from residue 788 to the C-terminus (residue 923) conferred PA-like incompatibility (see Methods) when transformed into C9-2 (*un*-*24*^OR^) (Figure 1B, bottom left). Omission of six amino acids from the C-terminus [hygunPA(861–917)] resulted in loss of incompatibility activity. Therefore, both specificity and incompatibility activity of UN-24^PA^ is encompassed in a 135 amino acid domain that corresponds to the flexible C-terminus arm of the large subunit contained within the RNR large subunit found in yeast [13,14].

Compared with *un*-*24*^PA^, a larger region of *un*-*24*^OR^ is required for incompatibility activity (Figure 1A). The construct hygunOR(788–929) did not carry incompatibility when transformed into C2(2)-1 (*un*-*24*^PA^). However hygunOR(335–929) caused OR-like incompatibility (see Methods), albeit to a lesser degree than the full length *un*-*24*^OR^ or the full length OR protein fused in frame with *hph* [hygunOR(Full), Figure 1A]. Deletion of 20 amino acids from the C-terminus [hygunOR(1–909)] of the full length UN-24^OR^ resulted in a loss of incompatibly activity. Taken together, these data showed that the C-terminus of both PA and OR is required for incompatibility and that activity regions differ for the two allelic forms.

### Expression of the PA incompatibility domain leads to an incompatibility-like reaction in yeast

**Figure 2:**
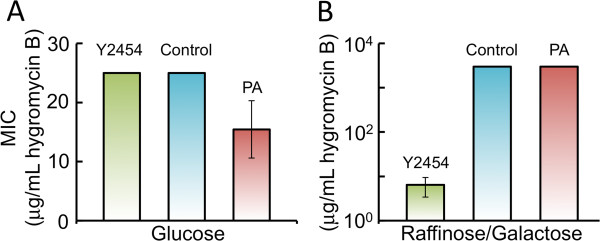
**Insertion of constructs into the *GAL1 *locus allows for control of trans**-**gene expression level. A**) We examined proper regulation of our constructs by assessing the minimum inhibitory concentration (MIC) of hygromycin B. When grown in medium containing glucose (YPD), the Y2454 wild-type and control yeast strains had similar MIC values that were significantly greater than that of the PA-expressing strain (P = 0.017). **B**) In contrast, when grown in medium containing galactose (YPRaf/Gal), a significantly higher MIC was observed for yeast strains containing *hph* constructs, as compared to the wild-type strain (P < 0.001) and there was no significant difference in MIC values of control and PA-expressing strains. Error bars in panels A and B indicate standard deviation based on 5 biological replicates.

In *N*. *crassa* it appears that *un*-*24*-associated incompatibility is due to a toxic interaction between the OR and PA protein forms [15]. However, analysis of the system is made difficult in *N*. *crassa* due to the presence of the *het*-*6* gene, which is tightly linked to and interacts with *un*-*24* during incompatibility reactions. Given that the amino acid sequence of ribonucleotide reductase is similar in *N*. *crassa* and yeast [10], that yeast apparently lacks a homolog to HET-6, and that yeast does not have an endogenous vegetative nonself recognition system, we explored whether the *un*-*24* incompatibility system could be transferred to yeast to provide further insight into the mechanism of *un*-*24*-associated incompatibility in general. We sought to determine if expression of the active *un*-*24* C-terminal domains [i.e., hygunPA(788–923) and hygunOR(335–929)] result in incompatibility-like phenotypes in yeast. We used homologous recombination to replace the *GAL1* coding region with our constructs and thus placed their expression under control of the *GAL1* promoter. Low or high level expression of our construct was obtained by growing the cells in medium containing glucose or galactose, respectively [16,17]. Four *GAL1* replacement strains were obtained in this way; a “control” strain with *hph* replacing *GAL1* (*GAL1*Δ::*hph*), a “PA” strain containing the hygunPA(788–923) incompatibility construct, and two “OR” strains containing either the hygunOR(788–929) or hygunOR(335–929). On Yeast-Peptone medium containing glucose (YPD), yeast that carried only *hph* exhibited the same hygromycin B MIC as the wild-type Y2454 strain (Figure 2A). When grown on Yeast-Peptone medium containing raffinose and galactose (YPRaf/Gal), all strains with *hph*-fused constructs exhibited a ~1000-fold increase in resistance to hygromycin B (Figure 2B). These results confirmed that our constructs were properly regulated in yeast. As evident in Figure 2A, growth on YPD revealed that low-level expression of the PA construct, but not OR (Additional file 1: Figure S1A and B), resulted in a significantly increased sensitivity to hygromycin B. This effect of the PA domain on yeast was interesting given its incompatibility function in *N*. *crassa* and was explored further.

**Figure 3:**
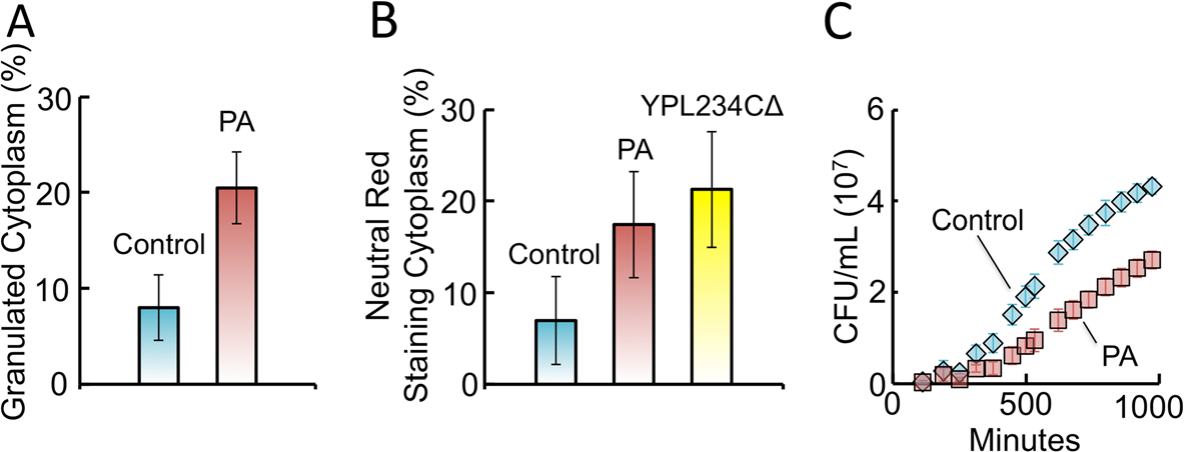
**Expression of the PA incompatibility domain at low**-**levels in yeast results in aberrant phenotypes. A**) Phase contrast microscopy revealed that PA-expressing yeast exhibit significantly more cells having a granulated cytoplasm compared to control strain (P = 0.007). Cytoplasmic granulation is a key feature of heterokaryon incompatibility in filamentous fungi. **B**) Significantly more PA-expressing yeast cells exhibit cytoplasmic acidification in comparison to control strain (P = 0.015) based on neutral red staining. The frequency of PA-expressing cells that exhibited an acidified cytoplasm did not differ from that of the vATPase-defective strain, YPL234C. For both **A** and **B**, error bars represent standard deviation based on 5 biological replicates where a minimum of 100 cells were counted. **C**) PA-expressing yeast had a slower growth rate in YPD compared to the control strain (P < 0.001). Growth was monitored by using a microplate reader and CFU was calculated from a standard curve of CFU versus OD_600_ (not shown). Error bars represent standard deviation based on three biological replicates.

Cytoplasmic granulation is one of the first recognizable cytological signs of heterokaryon incompatibility in filamentous fungi [18-20]. Consistent with this, phase contrast micrographs of PA-expressing yeast cells grown in YPD had significantly darker cytoplasmic granules when compared to the control strain (Figure 3A). We note that the contents of such granules are not known in yeast, nor are they known in *N*. *crassa*[18]. As incompatibility reactions progress in filamentous fungi, cytoplasmic vacuolization and ruptured vacuoles are observed, which can lead to cytoplasmic acidification [18,21]. We saw a similar phenotype in yeast using neutral red, a pH indicator dye that stains yeast vacuoles red [22], in that a significantly larger proportion of PA-expressing cells stained red throughout the cytoplasm than did control cells when growth was on YPD (Figure 3B). Overall, this staining pattern of the PA-expressing strain was indistinguishable from that of YPL234CΔ, a mutant yeast strain that lacks the vacuolar ATPase V0 domain subunit c’ and thus cannot effectively sequester H^+^ in the vacuole [23]. Therefore, neutral red staining indicated that, similar to the vATPase mutant strain, vacuolar membrane function is compromised in PA-expressing yeast strains. We also found that PA-expressing yeast grown on YPD had a significantly lower growth rate compared to the control strain (Figure 3C), a key characteristic of *un*-*24* incompatibility in *N*. *crassa*[15]. Interestingly, these aberrant yeast phenotypes were not evident when the PA construct was expressed at high levels on YPRaf/Gal (Additional file 1: Figure S2), nor were they observed when the OR constructs were expressed at low- or high-levels (Additional file 1: Figure S1C and D), suggesting that OR constructs did not confer incompatibility in yeast. In summary, low-level expression of PA in yeast caused three hallmark characteristics of fungal incompatibility: cytoplasmic granulation, perturbation of vacuole integrity, and growth inhibition.

### PA-expressing yeast have large cell volumes

**Figure 4:**
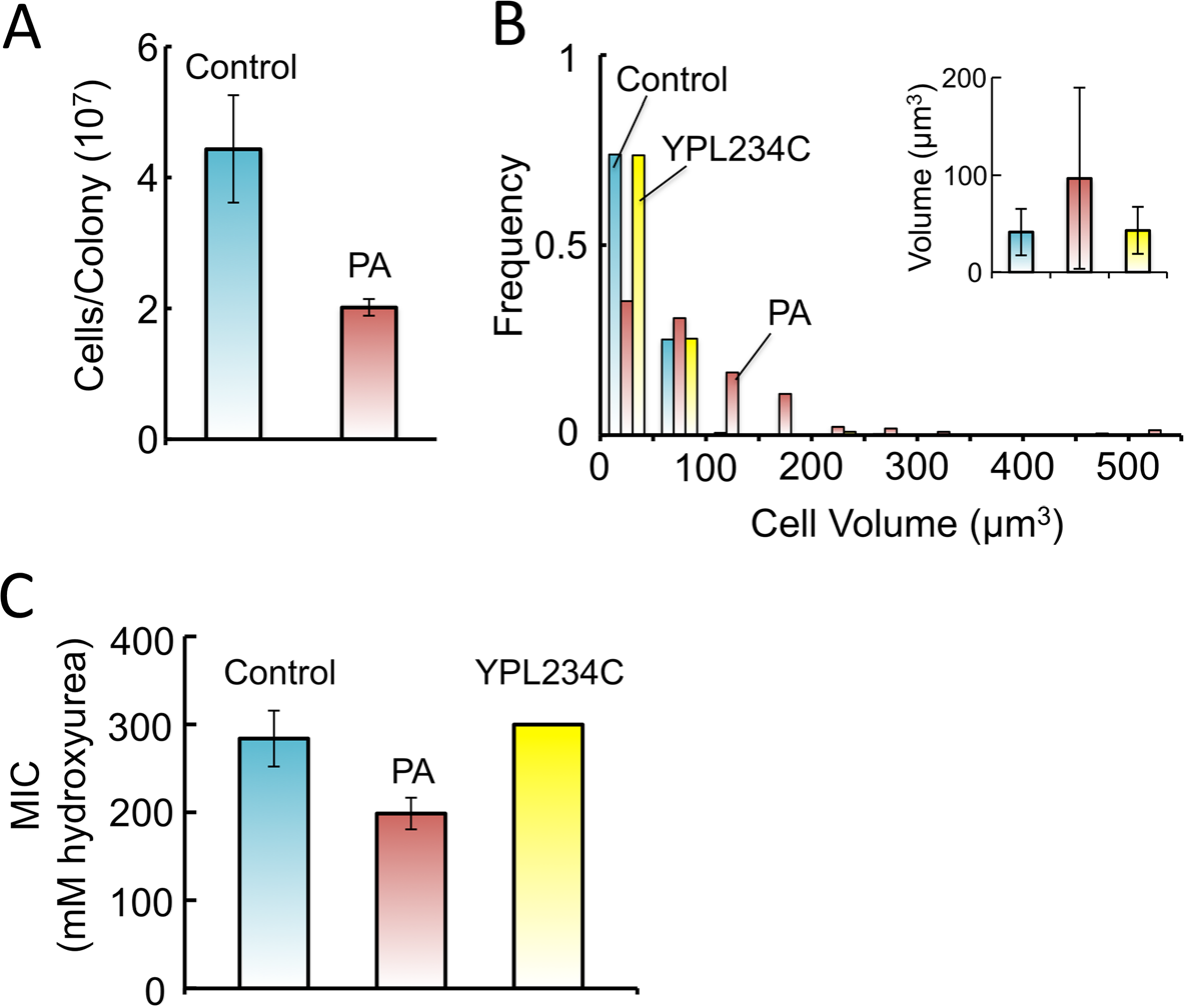
**Low**-**level expression of the PA incompatibility domain results in fewer and larger cells. A**) The number of cells in a 1 mm diameter colony was determined by cell counts with a haemocytometer. Significantly fewer cells were present in colonies of PA-expressing strain than the control strain (P = 0.003). Error bars represent standard deviation from 5 biological replicates. **B**) Volume measurements of cells grown on YPD show that PA-expressing cells were on average significantly larger than those of the control strain and the vacuolar ATPase mutant, YPL234C (P < 0.001, Wilcoxon/Kruskal Wallis test). Volumes were grouped into 50 μm^3^ bins and plotted. Inset: The average cell volume (±SD) of the three strains with shading as in main figure. **C**) PA-expressing yeast had increased sensitivity to hydroxyrurea, a potent inhibitor of RNR activity, when compared to the control strain and the vATPase-defective strain YPL234C, as determined through MIC measurements. Error bars represent standard deviation of 5 biological replicates.

An emerging theme in fungal nonself recognition is that incompatibility reactions involve lethal or detrimental protein complex formation between allelic or non-allelic proteins [15,24]. In *N*. *crassa*, it is hypothesized that a toxic UN-24-HET-6 complex mediates a strong incompatibility reaction, which often results in cell death [15]. In the absence of *het*-*6*, it is observed that an interaction between the PA and OR forms of UN-24 leads to a weak incompatibility reaction, characterized by an aberrant morphology and a significantly slower growth rate [15]. Since it appeared that the PA incompatibility domain was capable of causing an incompatibility-like reaction in yeast, we hypothesized that it might interact, and possibility interfere, with the yeast homolog RNR1 (Rnr1p) function. One prominent observation in yeast that lack Rnr1p, or that contain loss-of-function mutations in Rnr1p, is that they have significantly larger cell volumes [13,25]. Therefore, it may be expected that the interruption of RNR activity in yeast by the PA protein (PAp) would result in an increase in average cell volume. In support of this we initially observed that fewer colonies resulted from streaking a single PA-expressing colony on YPD plates (not shown). From cell counts with a haemocytometer, we found that equivalent sized 1 mm colonies of PA-expressing yeast contained significantly fewer cells than did control colonies (Figure 4A). We determined that this decrease in the number of cells per colony for the PA-expressing strain was not due to a reduction in viable cells based on Evan’s Blue vital staining (Additional file 1: Figure S3). Furthermore, as determined by microscopy, when grown in YPD, PA-expressing yeast had significantly larger cell volumes compared to the control strain and YPL234CΔ, the vATPase mutant strain discussed previously (Figure 4B), whereas cell volume distributions for the control strain and YPL234CΔ did not differ. We infer that the increased cell volumes of PA-expressing yeast were independent of cytoplasmic acidification.

The noted changes in cell volume are consistent with the hypothesis that PAp interferes with yeast Rnr1p function. Additional support for this idea came from our observation that PA-expressing yeast had an increased sensitivity to hydroxyurea over the control strain when grown in YPD (Figure 4C) but not on YPRaf/Gal (Additional file 1: Figure S4). Since hydroxyurea is a potent and specific inhibitor of RNR catalytic activity [26], this increased sensitivity to hydroxyurea provided further indications that low-level expression of PAp interferes with Rnr1p functions in yeast.

### PA-expressing strains contain a non-reducible PAp-Rnr1p protein complex

Immunoblotting methods were used to determine whether PAp binds to the yeast Rnr1p. Previously, it was reported that the oxidation state of yeast Rnr1p can be determined by SDS-PAGE [27]. In yeast, the RNR holoenzyme uses free radical chemistry to generate dNDPs from the respective NDPs. During 2^′^ hydroxyl group removal from the ribose moiety of the NDP, a disulphide bridge is formed between two cysteine residues in the catalytic site of Rnr1p. Once the newly formed dNDP is released from the catalytic site, the flexible C-terminus of the adjacent R1 subunit enters into the catalytic site and the disulphide bridge in the catalytic site is transferred to two cysteine residues located on the flexible C-terminus. The C-terminus arm then swings out of the catalytic site and this disulphide bridge is finally reduced by glutaredoxin or thioredoxin to reactivate the RNR holoenzyme [8,9]. When examined using SDS-PAGE, non-reducing conditions cause Rnr1p to resolve as two bands: the top band (lower mobility) represents the oxidized form (i.e., having a disulphide bridge between cysteine residues at the catalytic site) and the lower, high-mobility band represents the reduced form. When proteins are extracted under reducing conditions, only the lower band of reduced Rnr1p is evident [27].

**Figure 5:**
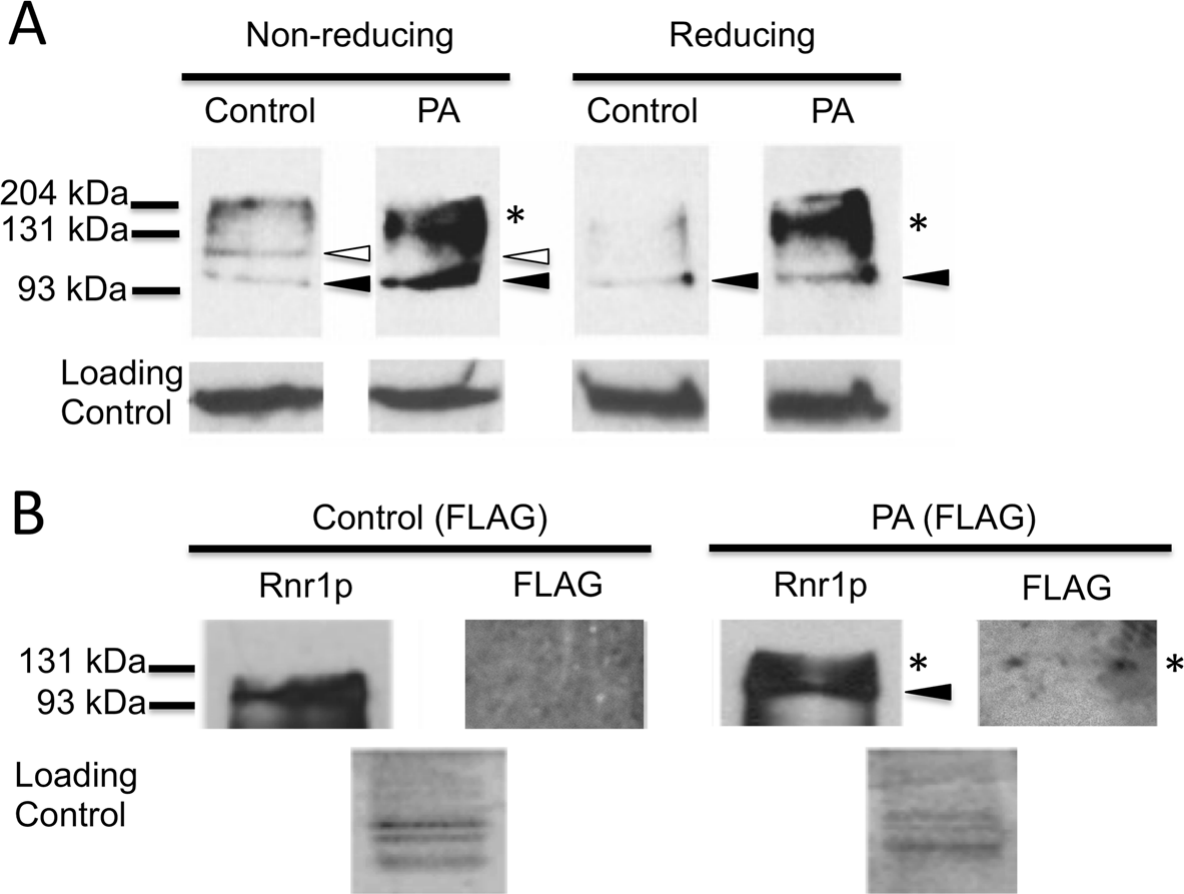
**The PA incompatibility domain interacts with yeast Rnr1p. A**) Proteins were extracted from PA-expressing and control yeast cells grown in YPD. Under non-reducing conditions, proteins extracted from PA-expressing yeast contained a lower amount of oxidized (open arrow) Rnr1p and a greater amount of reduced Rnr1p (solid arrow) compared to the control strain. As expected, oxidized Rnr1p in control strain is converted to the reduced form when proteins are extracted under reducing conditions. An intense band at 155 kDa (*), inferred to be a non-reducible PA (FLAG)p-Rnr1p complex (see Panel **B**), was observed in proteins extracted from PA(FLAG)-expressing yeast. Equal loading across lanes was based on Bradford assays and verified by a non-specified protein that reacted with the anti-Rnr1p polyclonal antibody (loading control). The images shown here are taken from one blot and as such exposure times are the same across all lanes. Similar results were observed in three independent experiments. **B**) Proteins were extracted under native conditions from PA-expressing and control yeast grown in YPD and subjected to size exclusion chromatography. Following fractionation, proteins were precipitated and concentrated, and treated with reducing agents before use in immunoblots. Co-fractionation and co-localization of the PA(FLAG)p (detected by anti-FLAG antibodies) and Rnr1p (detected by anti-Rnr1p antibodies) provides evidence for a 155 kDa PA(FLAG)p-Rnr1p complex (*) in Fraction 3 of the PA(FLAG) strain but not the control. Note that the range of proteins included in Fraction 3 is from 238 kDa to 55 kDa as determined by the elution of a HiMark pre-stained HMW Protein Standard (Invitrogen, not shown). Solid arrow indicates reduced form of Rnr1p. Equal loading was confirmed using a Coomassie stained duplicate gels. Molecular size markers are indicated at the left in both panels.

We found that under non-reducing conditions (no DTT or β-mercaptoethanol) Rnr1p from the control strain grown on YPD was resolved on immunoblots into reduced Rnr1p and oxidized Rnr1p (Figure 5A). In contrast, protein extracts of PA-expressing yeast showed the reduced form of Rnr1p (100 kDa), but little or none of the oxidized form. Interestingly, an intense band of ~155 kDa, the expected size of a complex consisting of PAp (55 kDa) and Rnr1p, was also observed from the PA-expressing yeast strain. When protein was extracted under reducing conditions (with DTT and β-mercaptoethanol) the band corresponding to oxidized Rnr1p was absent in both strains while both strains exhibited the reduced form of Rnr1p. Furthermore, the 155 kDa band that putatively represented the complex of PAp and Rnr1p remained present under these strong reducing conditions. Proteins extracted from the control and PA-expressing strains grown in YPRaf/Gal medium had no observable differences in the total amount of Rnr1p or the ratio of reduced to oxidized Rnr1p under reducing or non-reducing protein extraction conditions (Additional file 1: Figure S5). In addition, the ~155 kDa band was absent from extracts of both strains grown in YPRaf/Gal medium. Note that we verified the molecular weight of the oxidized and reduced Rnr1p bands using a strain that overexpresses Rnr1p (Additional file 1: Figure S5). These results indicated that a non-reducible PAp-Rnr1p complex is formed, but only when PAp is expressed at low levels.

To test whether the 155 kDa signal comprises Rnr1p and PAp, we subjected native-form proteins to size exclusion chromatography. For immunodetection, the FLAG epitope was inserted between *hph* and the PA incompatibility domain and this construct was inserted into *GAL1* to obtain the yeast strain “PA(FLAG)”. We verified that this strain exhibited incompatibility-like activity when grown on YPD medium (Additional file 1: Figure S6). As a control, we inserted the FLAG epitope after the *hph* gene, and obtained a “control (FLAG)” strain. When proteins were extracted from control(FLAG) and PA(FLAG) yeast grown in YPD and subjected to size exclusion chromatography, Rnr1p was detected predominantly in fraction 3 (elution range of 238 kDa - 55 kDa). The 155 kDa signal that putatively represents a complex of the PA(FLAG) protein [PA(FLAG)p] and Rnr1p was detected in fraction 3 and, consistent with previous results, was only observed in proteins extracted from the PA(FLAG)-expressing strain. When probed with anti-FLAG antibodies, the FLAG signal was not detected in fractionated proteins extracted from the control(FLAG) strain but was visible in fraction 3 from the PA(FLAG) yeast (Figure 5B). We note that this band was weak in intensity. However, this would be expected as expression from the *GAL1* promoter is minimal in the presence of glucose (i.e., ~ 150 fold lower than in the presence of galactose alone) and results in very low-levels of *GAL1* regulated protein [17]. Furthermore, we note that multiple attempts to pull down this complex using a variety of techniques (e.g., immunoprecipitation, affinity columns) were not successful. Nevertheless, these results suggested that the 155 kDa signal was composed of both yeast Rnr1p and the PA incompatibility domain. Interestingly, only PA(FLAG)p, and not the control(FLAG) protein, could be detected during low-level expression using anti-FLAG antibody. This suggests that PA(FLAG)p was being sequestered within this complex, effectively increasing its overall concentration in the cell.

### PA(FLAG)p interacts with Ssa1p, an Hsp70 protein, when PA(FLAG)p is over-expressed

**Figure 6:**
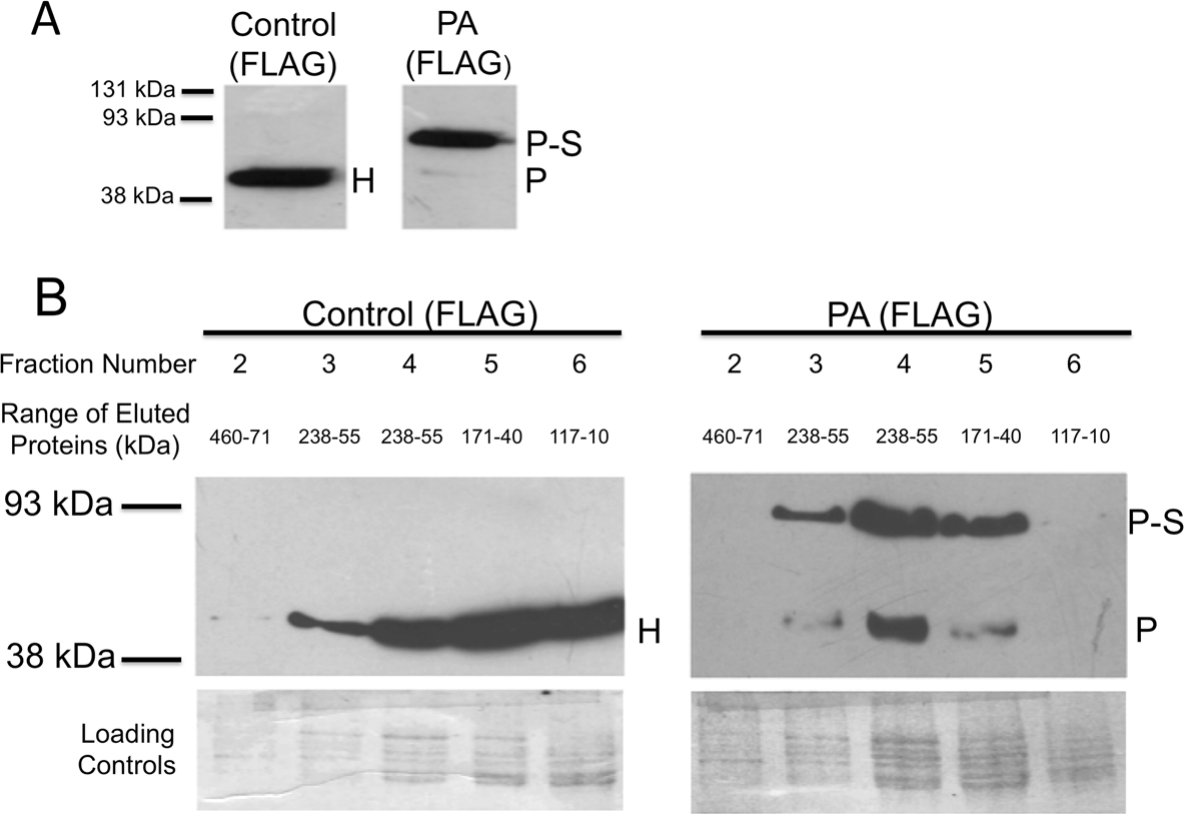
**High**-**level expression of the PA incompatibility domain results in an interaction with Hsp70 protein concomitant with remediation of aberrant PA**-**associated phenotypes. A**) Proteins were extracted under reducing conditions from PA-expressing and control yeast grown in YPRaf/Gal. Immunoblotting using anti-FLAG antibody reveals that over-expressed PA(FLAG)p forms a complex (P-S) with another protein that was identified by mass spectroscopy as Ssa1p (Additional file 1: Table S1). The weak PA(FLAG)p signal (P) demonstrated that most PA(FLAG)p is sequestered into this PA(FLAG)p-Ssa1p complex. The position of control (FLAG) protein is indicated (H). **B**) When overexpressed, virtually all of the PA(FLAG)p interacts with Ssa1p. Cells were grown overnight at 30°C in YPD, washed in PBS, resuspended in YPRaf/Gal and grown with shaking until mid-log phase. Proteins were then extracted and subjected to size exclusion chromatography as described in the main text. The control (FLAG) protein was detected in fractions 3–8. In contrast, the PAp monomer was detected only in the presence of the Ssa1p-PA(FLAG)p complex (fractions 3–5). This indicates that the majority of PA(FLAG)p was bound to Ssa1p and that treatment with reducing agents prior to immunoblotting dissociated some but not all of the PA(FLAG)p from the complex. Duplicate Coomassie blue stained protein gels were used to verify equal loading across lanes. Positions of molecular weight markers are shown at left. For both panels, similar trends were observed in two independent extractions and immunoblots.

We investigated the counterintuitive observations noted earlier that PAp expressed at low (on YPD), but not at high-levels (on YPRaf/Gal), caused incompatibility-like symptoms in yeast. Immunoblots were done with proteins extracted under reducing conditions from PA(FLAG) and control(FLAG) yeast grown in YPRaf/Gal (Figure 6A). Using anti-FLAG antibody, we observed a ~41 kDa signal in the control strain, which corresponds to the control(FLAG) fusion protein, and two bands of ~55 and ~85 kDa in the PA(FLAG) strain. The smaller of these latter two proteins is the expected size of PA(FLAG)p while the larger protein was immunopurified and identified by mass spectroscopy to contain sequences of Ssa1p, an Hsp70 homolog (Additional file 2: Table S1). We concluded that this band is a complex formed between Ssa1p and PA(FLAG)p since it was larger than the expected mass of Ssa1p (70 kDa) and binds to anti-FLAG antibodies. We note that the majority of PA(FLAG)p in YPRaf/Gal-grown cells was associated with Ssa1p since the ~85 kDa signal was significantly more intense than the ~55 kDa signal. Interestingly, size exclusion chromatography showed that PA(FLAG)p is only in fractions that contain Ssa1p indicating that nearly all of the detectable PA(FLAG)p was complexed with Ssa1p (Figure 6B). This PA(FLAG)p-Ssa1p complex is quite stable since treatment with reducing agents liberated some, but not all PA(FLAG)p from the Ssa1p complex. Furthermore, in a strain with *SSA1* deleted, different chaperone proteins, Ssb2p, or Hsp60 (both detected in our analysis) tightly complexed with the PA(FLAG)p (Additional file 1: Figure S7, Additional file 2: Table S2). We note that several Hsp70 proteins, including both Ssa1p and Ssb2p, assist in protein folding [28] and have been observed to interact with aggregating proteins [29,30]. Therefore, it appears that Ssa1p and Ssb2p/Hsp60 effectively bind to the PAp incompatibility factor when it is overexpressed in yeast.

## Discussion

We define a protein domain with incompatibility function in RNR from *N*. *crassa* and demonstrate it can elicit an incompatibility-like reaction in yeast. Previous studies have examined trans-species expression of fungal nonself recognition genes in closely related filamentous fungi [31-33]. In particular, expression of *N*. *crassa tol* results in *mat*-associated heterokaryon incompatibility in *Neurospora tetrasperma*[34], and PA alleles of *N*. *crassa het*-*c* appear to produce growth defects reminiscent of heterokaryon incompatibility in *Podospora anserina*[31] and *Aspergillus niger*[33]. Unlike these previous studies, we extended the confirmation of incompatibility activity to a functional analysis of the *un*-*24* nonself recognition system, initiating an understanding of its mechanisms. Interestingly, and unlike the filamentous fungi, a vegetative incompatibility system has not been described in yeast and *in silico* experiments showed that yeast lacks homologs to several heterokaryon incompatibility domains found in filamentous ascomycete fungi [12]. Nevertheless, our work shows that such a system can operate in yeast. As demonstrated here, heterologous expression of nonself recognition factors in yeast can also lead to fundamental insights into mechanisms of activity and control of nonself recognition factors. In such a system, core interactions of incompatibility domains can be studied without a confounding effect of other potentially interacting incompatibility factors. In the future, it would be interesting to determine if these incompatibility reactions can be enhanced in the yeast system by the addition of other known incompatibility factors from *N*. *crassa*. For example, it is known that the allelic *un*-*24* incompatibility in *N*. *crassa* is significantly strengthened by non-allelic interactions with *het*-*6* factors [15].

One emerging trend observed with heterokaryon incompatibility systems is the involvement of protein-protein interactions that trigger cell death. This is particularly evident in the *het*-*c* system of *N*. *crassa*[35] and the *het*-*s* system in *P*. *anserina*[24]. Our results indicate that *un*-*24*-associated incompatibility is likewise mediated by protein interactions. When expressed at low levels, the PAp domain apparently forms a complex with Rnr1p that results in incompatibility-like phenotypes in yeast. The observed “toxicity” of the Rnr1p-PAp complex in yeast is consistent with incompatibility associated with coexpression of PA and OR alleles of *un*-*24* in *N*. *crassa*[15] and with a recently published study that demonstrates that the C-terminus of *un*-*24*^PA^ is able to form a non-reducible complex with UN-24^OR^ in *N*. *crassa*, the presence of which is correlated with incompatibility [36]. Unlike *N*. *crassa* where there is a single gene (*un*-*24*) encoding the RNR large subunit, yeast contains the paralogs *RNR1* and *RNR3*; *RNR1* is cell-cycle regulated and used under normal cellular growth, and *RNR3* is upregulated in response to DNA damage [37]. The partial redundancy of Rnr1p and Rnr3p may alleviate some toxic effects of expressing PAp in yeast. Interestingly, yeast that lack *RNR1* have reduced vacuolar ATPase activity [38], have increased sensitivity to antifungal agents [38,39] and to hydroxyurea [40], have an increased cell size [41] and have a longer cell cycle [27] (which may lead to an decreased overall growth rate) -- all of which are consistent with the effects of PAp expression in yeast that are presented herein. The association of an Rnr1p-PAp complex with several incompatibility-like phenotypes suggests that PAp incompatibility activity operates in yeast through a loss or reduction in RNR catalytic function, a hypothesis that is consistent with the endogenous activity of UN-24 that should now be examined closely in *N*. *crassa*.

Our insights on trans-species activity of PAp in yeast may have a bearing on two other interesting characteristics of incompatibility systems in filamentous fungi. Specifically, that Hsp70 proteins alleviate PAp-associated incompatibility in yeast may suggest that chaperones have roles in the “escape” process, and in suppressing heterokaryon incompatibility in stages leading up to and during the sexual cycle [42]. Escape is defined as a sudden shift from the incompatible state (aberrant colony and cell morphologies and slow growth rate) to a wild-type morphology and growth rate [43]. The mechanism of escape is often correlated with large deletions, rearrangements and other mutations of incompatibility genes [43-46]. Likewise, how multiple incompatibility genes in filamentous fungi are inactivated during the sexual cycle is a mystery that may be generally relevant to a dampening of nonself recognition to permit zygote development within the mother in other sexually reproducing organisms. Along this line, some heat shock proteins are specifically expressed in perithecia and in unfertilized sexual tissues in *N*. *crassa*[47,48].

It is interesting to note that, in addition to functioning as chaperone proteins, Hsp70 family members are upregulated during cellular stress and can bind to and facilitate degradation of toxic, abnormal protein complexes [29,49-51]. We surmise that alleviation of incompatibility-like phenotypes upon PAp overexpression in yeast may occur through two mechanisms. First, Ssa1p has been observed to sequester toxic protein precursors in yeast to prevent them from aggregating [52]. Therefore, it is possible that, upon high-level expression, PAp is specifically targeted by Ssa1p prior to its interaction with Rnr1p and that low-level expression of PAp is insufficient to trigger Ssa1p for sequestration but sufficient enough to result in toxicity. Secondly, Ssa1p may assist in the degradation of non-reducible PAp-Rnr1p complexes. Ssa1p has been shown to interact with partially degraded protein aggregates [29] and has been implicated in transferring misfolded proteins to the yeast proteasome for degradation [53-56]. It should be noted, however, that the amount of non-complexed PAp observed in Figure 6 should be sufficient (as compared to the intensity of the band observed in Figure 5) to cause the incompatibility-like phenotypes. As with other instances where heat shock proteins interact with and/or degrade toxic protein complexes, it is likely that the mechanism by which Ssa1p alleviates the toxicity of PAp is more complex than the simple explanations offered above. As such, we currently do not have a robust model for the role of Ssa1p in modulating PAp toxicity. Nevertheless, the clear correlation we observe between the formation of a Ssa1p-PAp complex and the amelioration of incompatibility-like phenotypes in yeast poses questions about whether Hsp70 proteins play a role in escape and in modulating incompatibility during the sexual cycle in *N*. *crassa* and other organisms.

Finally, due to its essential function and evolutionary conserved structure, type I RNRs represent attractive drug targets. Indeed, the development of peptide inhibitors that disrupt the quaternary structure or activity of RNR is a field that may present a relatively safe and efficacious chemotherapeutic strategy [57]. Ironically, inherent within the *N*. *crassa* large subunit of RNR already lies the potential for a strain-specific antibiotic-like activity, as manifested by the growth inhibition of cells resulting from nonself fusions in *N*. *crassa*. The trans-species inhibitory activity of PAp in yeast further suggests that the *un*-*24* incompatibility domain may present insights into a broad-spectrum antimicrobial peptide that can be targeted to selected species or strains.

## Conclusions

We have described a novel nonself recognition domain located in the C-terminus of UN-24. Our results demonstrated that the PA, but not the OR, C-terminus retains activity when expressed in *S*. *cerevisiae*. We demonstrate that low-level expression of PA(p) results in several incompatibility-like cytologies, an increase in cell size and the formation of a complex consisting of yeast Rnr1p and PA(p). These phenomena are resolved when PA(p) is expressed at high level, where an apparent complex between Ssa1p and PA(p) forms. Results from our study indicate that yeast can be used to investigate nonself recognition systems. Furthermore, our study shows that Hsp70 proteins can alleviate incompatibility, which may suggest their involvement in the escape process or in the sexual cycle of *N*. *crassa*. Finally, given the unique trans-species activity of the PA(p) protein, and the ubiquitous and evolutionarily well conserved target, RNR, it would be interesting to determine if variations of this protein have applications as chemotherapeutic agents.

## Methods

### Manipulation of N. crassa strains and molecular genetic methods

The *N*. *crassa* strains used [with Oakridge (OR) alleles at all undesignated *het* loci] were: C2(2)-1 (*un*-*24*^PA ^*het*-*6*^PA ^*thr*-*2 a*) and C9-2 (*un*-*24*^OR ^*het*-*6*^OR ^*het*-*c*^PA ^*thr*-*2 a*). DNA cloning was done with plasmids pCB1004 [58], which contains the hygromycin phosphotransferase (*hph*) selectable marker conferring hygromycin resistance, and pCR2.1 (Invitrogen, Carlsbad, CA). PCR reactions were performed with *Taq* DNA polymerase (New England Biolabs, Mississauga, ON) or iProof DNA polymerase (BioRad, Mississauga, ON) according to the manufacturer’s recommendations. Oligonucleotide primer sequences are available upon request. All constructs were sequenced and verified as error-free. DNA transformation and spheroplast preparation of *N*. *crassa* strains were done as previously described [10]. *N*. *crassa* transformants were selected on medium containing 200 μg/ml of hygromycin B (Roche, Mississauga, ON).

#### *un*-*24* constructs used in incompatibility assays

The *un*-*24*^OR^ or *un*-*24*^PA^ portions of the fusion genes were derived from standard *N*. *crassa* strains as described above. Fragments of *un*-*24* were amplified with a forward primer that introduced a *Spe*I site allowing for an in-frame fusion with the *hph* marker, and a reverse primer that introduced a stop codon (or spanned the resident stop codon of *un*-*24*) as well as a flanking *Eco*RI site. All amplicons were cloned into pCR2.1. *Eco*RI and *Spe*I were then used to cut out the *un*-*24* fragment and *Bgl*II and *Spe*I were used to cut out the *hph* fragment. The digests were heat-inactivated, mixed and ligated before PCR amplification using the primer that binds to the *hph* promoter and the appropriate *un*-*24* reverse primer. The *hph*-*un*-*24* fusion products were then cloned into pCR2.1.

Our criteria for identifying incompatibility activity of OR and Panama (PA) constructs in *N*. *crassa* varies in accordance with the asymmetry of the system [15]. We recognized *un*-*24*^OR^-associated incompatibility activity by a significant decrease (~95%) in the number of viable colonies generated when the *un*-*24*^OR^ allele is transformed into the *un*-*24*^PA^ strain, in comparison to transformations with the vector control. In contrast, when *un*-*24*^PA^ is transformed into the *un*-*24*^OR^ strain, there is a modest (~20%) reduction in number of transformants recovered. However, 50 – 90% of the transformant colonies are small and have an irregular “star-like” growth form that contrasts with the wild-type “cloud-like” form of compatible transformants. Subcultures of the star-like colonies exhibit a self-incompatible phenotype as recognized by a slow growth rate and few aerial hyphae or conidia. This self-incompatible phenotype is inherently unstable and will spontaneously convert after about one week of continuous growth to near wild-type growth rate and morphology, a phenomenon called “escape” [11]. Therefore, to recognize *un*-*24*^PA^-associated incompatibility activity we used three criteria: 1) more than half of colonies on the transformation plates displayed the self-incompatible morphology, 2) subcultures of these colonies had growth rates that were more than ten times lower than those of wild-type colonies and, 3) these subcultures subsequently escaped to a wild-type morphology and growth rate. Constructs were tested for incompatibility activity in at least three separate trials using transformation assays with strains C9-2 and C2(2)-1.

### Yeast Strains, media and growth conditions

*S*. *cerevisiae* strains used in this study were derived from those listed in Additional file 2: Table S3 and were cultured by standard methods [59]. Selective plating of yeast transformants was performed with 100 μg/ml hygromycin B or 100 μg/ml nourseothricin (Werner Bioagents, Jena, Germany). The *hph*-*un*-*24* fusion constructs were inserted into the *GAL1*(YBR020W) locus of strains Y2454 (*un*-*24*^PA^ constructs) or Y3068 (*un*-*24*^OR^ constructs) by homologous recombination using lithium acetate transformation [60]. Correct insertion of *hph*-*un*-*24* constructs were confirmed by yeast genomic DNA extraction [61] and PCR amplification with primers that flank *GAL1*.

The PA(FLAG) construct was made by fusing a standard FLAG epitope in-frame between *hph* and the *un*-*24*^PA^ incompatibility domain. The control(FLAG) construct was made by in-frame fusion of the FLAG epitope to the 3^′^ end of *hph*. Strains that carried these FLAG constructs in a *SSA1* knockout background were obtained by mating YAL005CΔ (Additional file 2: Table S3) separately to yeast strains containing PA(FLAG) and control(FLAG) constructs, random sporulation [59], and selection of double mutants on 200 μg/mL G-418 (Bioshop, Oakville, ON) and hygromycin B.

### Microscopy, Growth Rate and Minimum Inhibitory Concentration (MIC)

Cells were examined by phase-contrast with a Zeiss Axiovision II microscope (Toronto, ON). Use of neutral red as a pH-sensitive stain was previously described [22]. The frequency of cells that had a red-stained cytoplasm (as opposed to those with a bright red central vacuole only) was determined using a double-blind approach. Cell size was determined as previously described [62] based on cell measurements taken from micrographs of randomly selected fields of view. The number of cells in 1 mm diameter colonies of similar height was determined by resuspending the colony in 0.1 M NaCl and cell counts using a haemocytometer. Minimum inhibitory concentration (MIC) values for hygromycin B and hydroxyurea (Bioshop, Lot#1932H) were determined using standard methods as previously described [63]. The MIC was recorded as the lowest concentration of inhibitor at which no growth was visible after 2 days incubation at 30°C.

### Detection of FLAG-tagged proteins and Rnr1p

Mid-log phase cells grown in YPRaf/Gal were harvested, washed once with ddH_2_O, and resuspended in either a) non-reducing extraction buffer [20 mM Tris HCl (pH 7.9), 10 mM MgCl2, 1 mM EDTA, 5% glycerol, 0.3 M ammonium sulphate, 1 mM PMSF and 1 Complete Mini-Protean tablet (Roche, Mississauga, ON)], or b) reducing buffer [20 mM Tris HCl (pH 7.9), 10 mM MgCl2,1 mM EDTA, 5% glycerol, 0.3 M ammonium sulphate, 10 mM DTT, 1 mM PMSF and 1 Complete Mini-Protean tablet]. Cells were lysed using 0.5 mm silica beads and 30 seconds of vigorous vortexing followed by cooling on ice for 2 minutes. This bead vortexing was repeated four times. Cell debris was removed through centrifugation at 16,000 × *g* for 1 hour at 4°C. Proteins were quantified using a Bradford assay. Cytosolic protein was combined with 2X Laemmli buffer (125 mM Tris HCl (pH 6.8), 20% glycerol, 4% SDS, 0.004% bromophenol blue, with or without 15.4 μg/mL DTT and 0.7 M β- mercaptoethanol), separated on 4-20% SDS-PAGE and immunoblotted using either anti-FLAG M2 peroxidase conjugate antibody (1:1000; Sigma-Aldrich, Oakville, ON) or a combination of anti-rabbit Rnr1p primary antibody (1:7500) that was kindly provided to us by Joanne Stubbe (Department of Chemistry, MIT, Cambridge, MA) and donkey, anti-rabbit secondary antibody (1:15000; Promega, Madison, WI).

### Immunoprecipitation

Cytosolic proteins were extracted as described above and captured using anti-FLAG M2 antibodies bound to agarose beads (Sigma-Aldrich). Unbound proteins were removed by washing the beads three times in 40 mM Tris–HCl (pH 8.0), 10 mM MgCl_2_, 20% glycerol, 0.2% Tween 20, 0.5 M KCl, 0.1% PMSF, 0.07% β-mercaptoethanol, and one Mini-Protean complete inhibitor tablet. Bound protein was eluted with 10 μg/ml FLAG peptide (Sigma Aldrich). SDS-PAGE and immunoblotting were performed as described above.

### Size exclusion chromatography

Proteins were extracted as described above under non-reducing conditions. The supernatant was removed, combined with 5 mg/ml of dextran blue 2000 (Pharmacia Corporation, North Peapack, NJ) and 5 mg/ml NiCl (BDH, Poole, England) and subjected to size exclusion chromatography (30 cm length, bed volume 25 ml; BioRad, Missassauga, ON) using Sephacryl 300 HR (Sigma Aldrich) pre-equilibrized in 0.1 M NaCl. Proteins were eluted with a flow rate of ~0.2 ml/min and collected in 1 ml fractions beginning with elution of dextran blue. Proteins were precipitated and concentrated using trichloroacetic acid (Sigma-Aldrich) and solubilized in 1% SDS, 9 M urea, 25 mM Tris–HCl pH 6.8, 1 mM EDTA by boiling for 10 minutes. SDS-PAGE and immunoblotting were performed as described above. Size range was determined by loading a HiMark Pre-Stained HMV Protein Standard (Invitrogen).

### LC-MS/MS Analysis

Affinity purified proteins were separated by SDS-PAGE and stained with Coomassie blue. Protein bands were excised and digested in the gel using trypsin. Mass spectroscopy was performed at the Ottawa Institute of Systems Biology (Ottawa, Ontario). Protein identity was determined using Mascot (Matrix Science Inc., Boston, MA).

### Statistical analysis

Unless otherwise noted, statistical significance was assessed using a two-tailed Student’s *T*-test. Values were determined to be statistically significant when P ≤ 0.05.

### Availability of supporting data

The supporting information contains Supporting Additional file 1: Figures S1-S7 and Supporting Additional file 2: Tables S1-S3.

## Abbreviations

PA: Panama; OR: Oakridge; RNR: Ribonucleotide reductase; (NDP): Ribonucleoside diphosphates; dNDPs: deoxyribonucleoside diphosphates; MIC: Minimum inhibitory concentration; YPD: Yeast peptone dextrose medium; YPRaf/Gal: Yeast peptone raffinose galactose medium.

## Competing interests

The authors declare that they have no competing interests.

## Authors’ contributions

RPS assisted in conceiving research, performed experiments, interpreted results and wrote the manuscript. KW performed experiments and interpreted results. MLS assisted in conceiving research, interpreted results and wrote the manuscript. All authors approved the manuscript.

## Supplementary Material

Additional file 1: Figure S1 In contrast to PA-expressing strains, yeast expressing the UN-24^OR^ incompatibility domain have no discernable incompatibility-like phenotypes (P > 0.35). Yeast strain Y3068 is the untransformed parental strain, the “control” strain has *hph* integrated into the *GAL1* locus, whereas hygunOR(788-929) and hygunOR(335-929) have OR incompatibility- domain replacements of GAL1. Cells were grown overnight at 30°C in YPD, washed in PBS, resuspended in YPD or YPRaf/Gal and grown with shaking until mid-log phase. Determination of MIC (A and B), granulated cytoplasm (C), and neutral red staining (D) were performed as described in the Methods section. Error bars indicate standard deviation from a minimum of 3 biological replicates for all panels. For both C and D a minimum of 100 cells were counted. **Figure S2.** Incompatibility-like phenotypes of control and PA strains were not significantly different when constructs were over-expressed by growing yeast in YPRaf/Gal (P > 0.05 in all cases). Briefly, cells were grown overnight at 30°C in YPD, washed in PBS, resuspended in YPRaf/Gal and incubated with shaking until mid-log phase. Cytoplasmic granulation (A), neutral red staining (B) and growth rate (C) analyses were performed as described in the Methods section. Error bars indicate standard deviation from 5 biological replicates. **Figure S3.** The frequency of dead cells tended to be greater in the strain over-expressing the PA construct than in the control strains, but did not significantly differ during lag, mid-log and stationary phase growth on YPD (P > 0.05 in all cases). Dead cells were recognized by deep blue color using the vital stain Evan’s Blue and light microscopy. OD^600^ was used to determine 2 growth phase based on the growth curve presented in Figure 3C. For vital staining, cultures were washed three times in PBS, resuspended in PBS, mixed with an equal volume of 1% w/v Evan’s Blue, held for 5 min at room temperature and examined at 40X using bright-field microscopy. A minimum of 100 cells was counted for each trial and three biological replicates were performed using a double-blind design. **Figure S4.** In YPRaf/Gal PA-expressing yeast had the same sensitivity to hydroxyrurea as the control strain (P = 1.0). Cells were grown overnight at 30°C in YPD, washed in PBS, resuspended in YPRaf/Gal and shaken until mid-log. The MICs of 5 biological replicates were measured as described in the Methods section. **Figure S5.** The ~155 kDa Rnr1p-PA(FLAG)p band was not present on immunoblots of yeast grown in YPRaf/Gal. Initially, we used a yeast strain that overexpressed Rnr1p (p*Gal*-RNR) when grown on galactose in order to verify the position of the oxidized and reduced forms of Rnr1p (left lane). We then extracted proteins from the control and the PA-expressing strains grown in YPRaf/Gal and immunoblotted them with anti-Rnr1p antibody as described in the main text. While Rnr1p was detected in the control and PA strains, the ~155 kDa band was markedly absent. The blot shown includes the range encompassing proteins or 155 kDa (i.e. from the 131 kDa molecular weight marker to the loading/running gel interface, as indicated). The same result was observed in two independent replicate experiments. **Figure S6.** Low-level expression of the PA(FLAG) construct was similar to the PA construct (P = 0.67) in causing *het*-associated cytoplasmic acidification, as determined by neutral red staining. Both PA-expressing strains had a higher frequency of cells exhibiting cytoplasmic acidification compared to the control (P < 0.05 in both cases). Neutral red staining was performed on 5 biological samples as described in the Methods section. **Figure S7.** When the PA construct was overexpressed in a strain with Ssa1 deleted the chaperone proteins Ssb2 and/or Hsp60 associate with PA(FLAG)p. We determined this by first crossing PA(FLAG)-expressing yeast with YAL005CΔ, an *SSA1* knockout strain, to obtain a PA(FLAG) *SSA1*Δ strain. This strain was grown to mid-log phase in YPRaf/Gal and proteins were extracted under non-reducing conditions. Anti-FLAG antibodies revealed an ~85 kDa band in immunoblots that was identified by mass spectroscopy to contain Ssb2p and Hsp60p (Additional file 2: Table S2, P-HSP). The 85 kDa protein is larger than expected for Ssb2p (67 kDa) or Hsp60p (61 kDa) and, since it was detected by anti-FLAG antibodies, likely represents a complex with PA(FLAG)p. Control(FLAG)p indicated with ‘H’. Click here for file

Additional file 2: Table S1 Mascot results of anti-FLAG purified protein bands from hygFLAGunPA-expressing yeast grown in YPRaf/Gal. The ~54 kDa and ~85 kDa protein bands generated peptide sequences that corresponded to hygromycin phosphotransferase protein and Ssa1p, respectively. **Table S2.** Mascot results of anti-FLAG purified protein from yeast that lacked *SSA1* and that expressed hygFLAGunPA. The ~ 85 kDa protein band yielded peptides that corresponded to the mitochondrial chaperone Hsp60 and to the cytosolic Hsp70 homolog, Ssb2p. **Table S3.** Yeast strains used in this study. Click here for file
