# Supplementary figures and images for: Trans-species activity of a nonself recognition domain
Source: BMC Microbiol. 2013 Mar 22;13:63. doi: 10.1186/1471-2180-13-63 (PMC3618301; doi:10.1186/1471-2180-13-63)

Figure S1

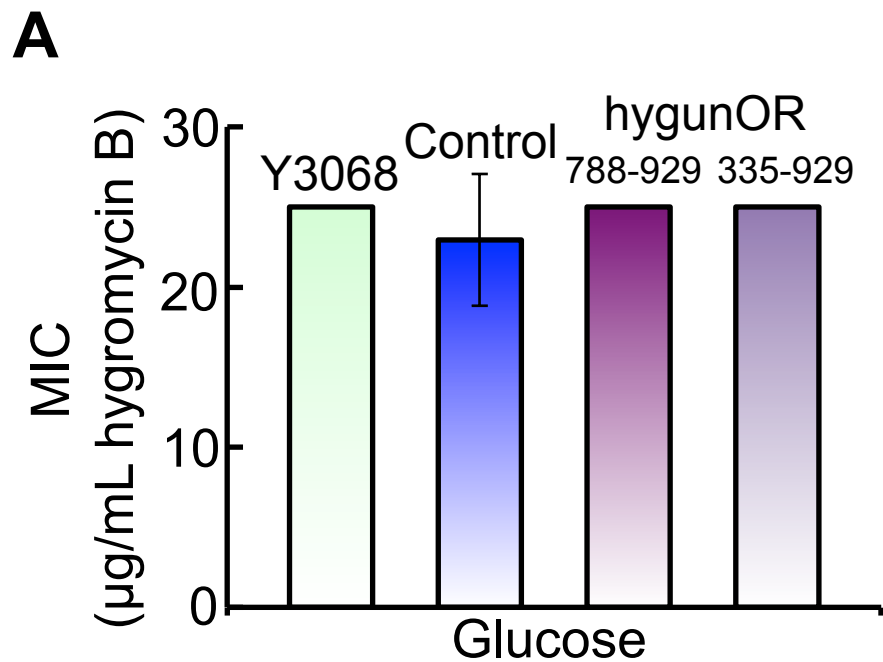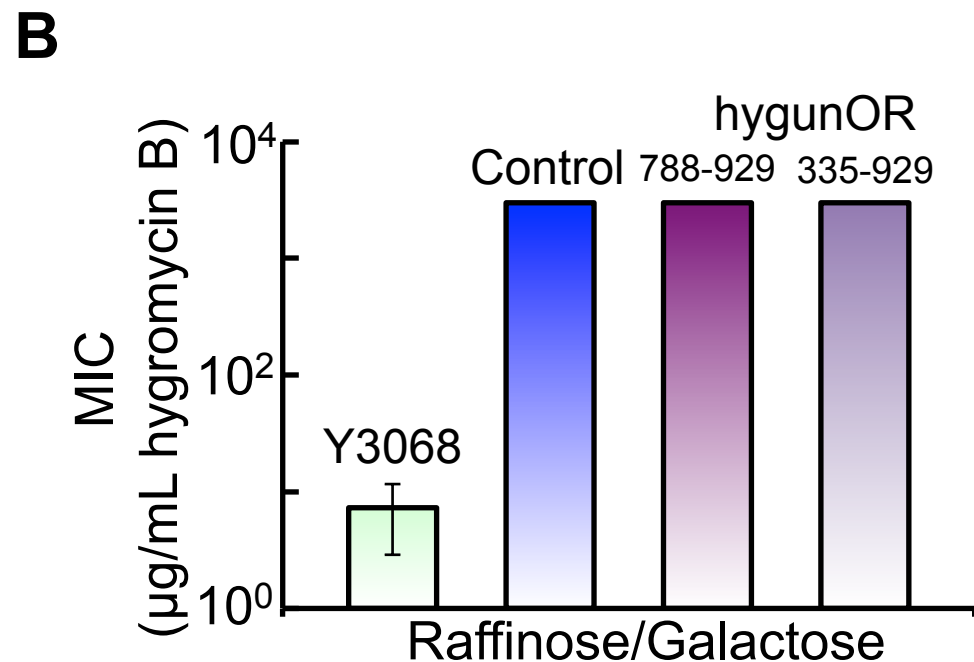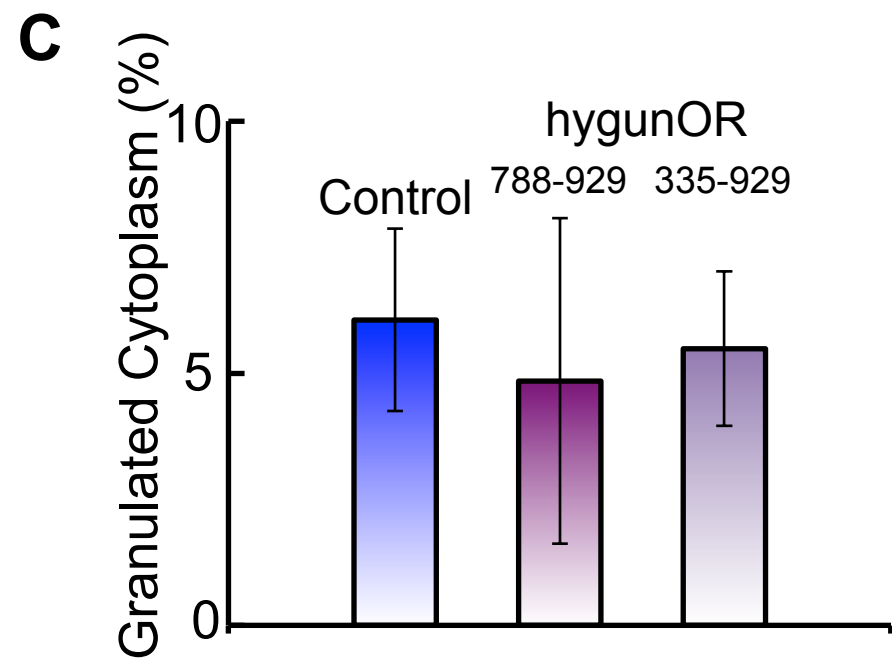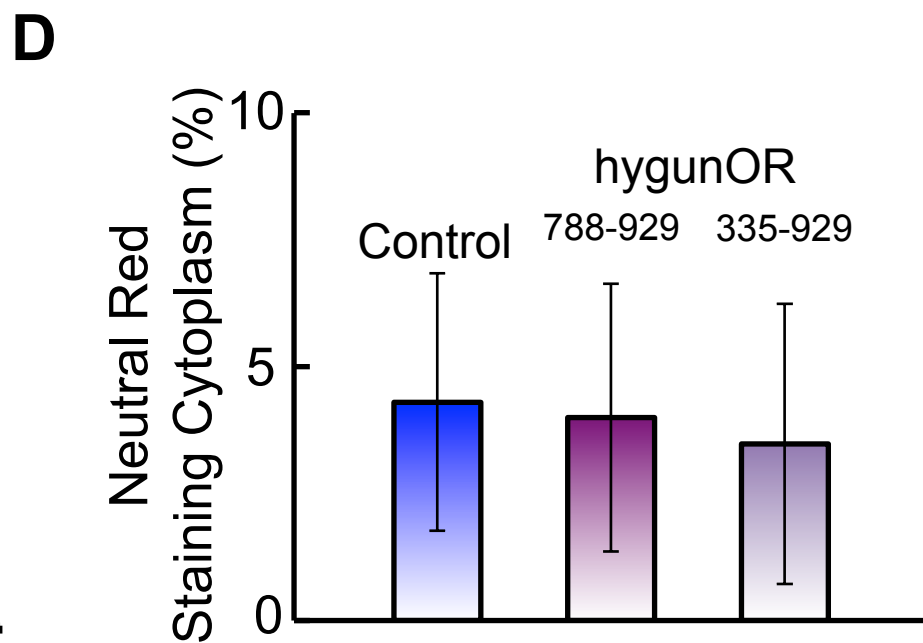

Figure S2

**A**

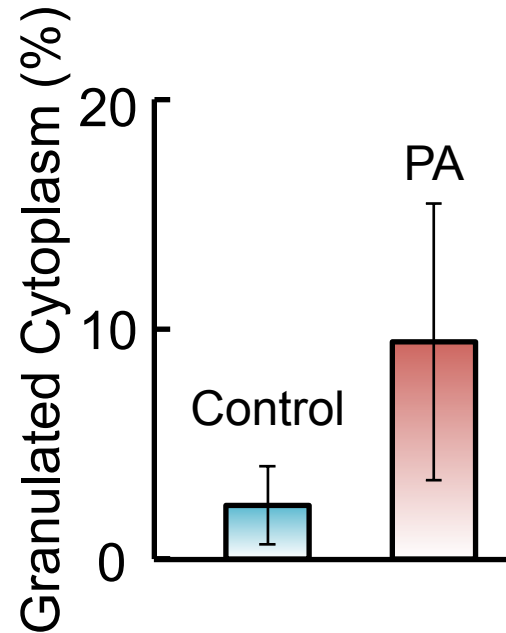

**B**

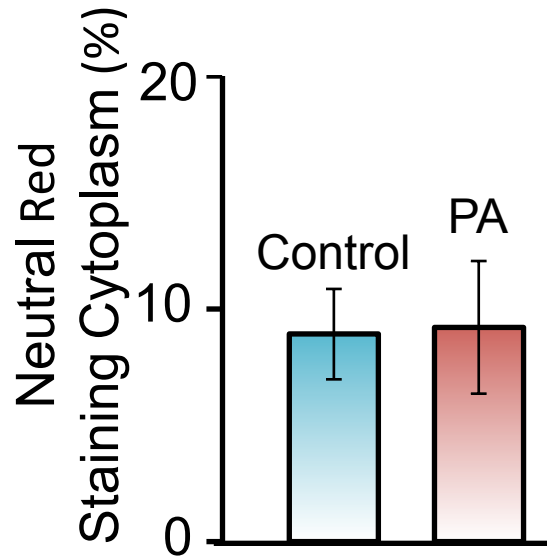

**C**

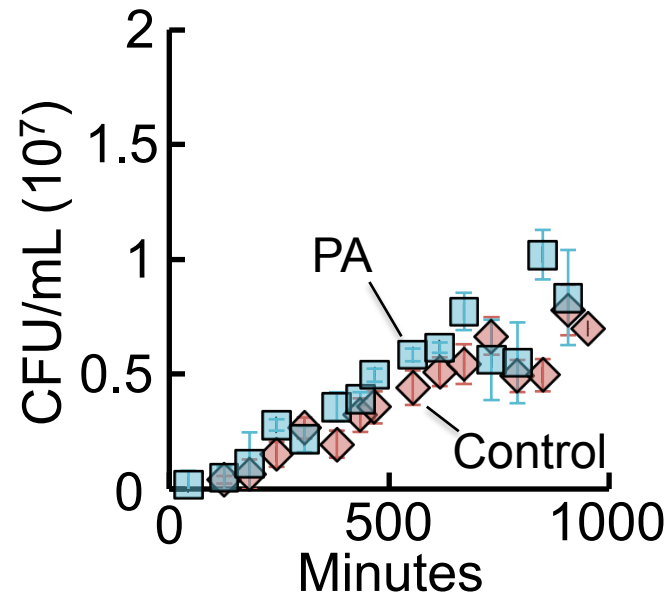

Figure S3

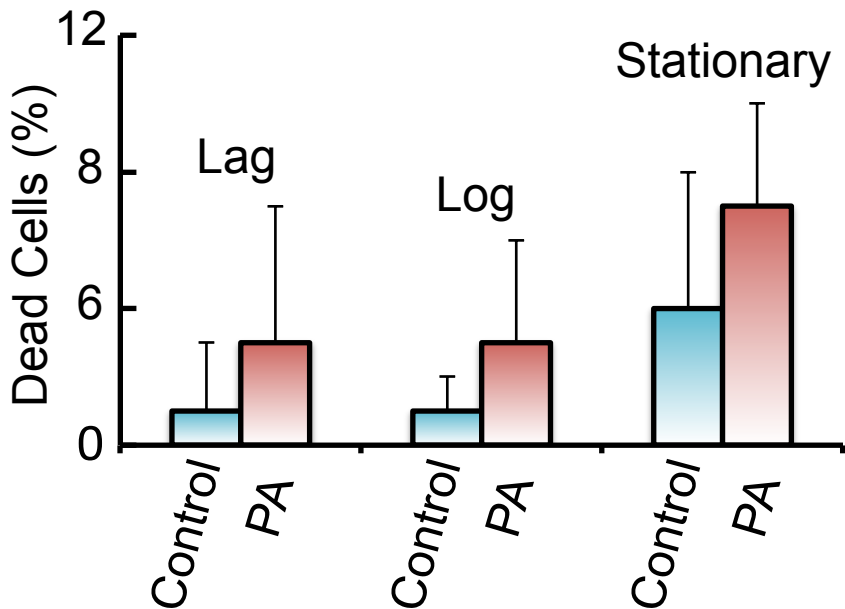

Figure S4

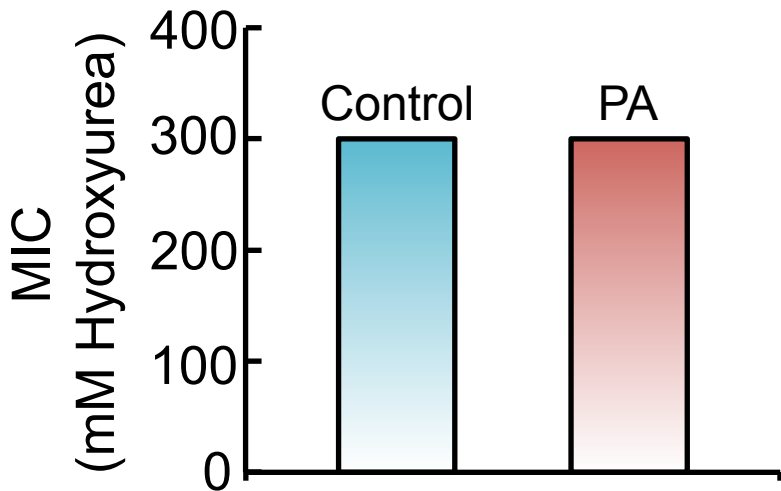

Figure S5

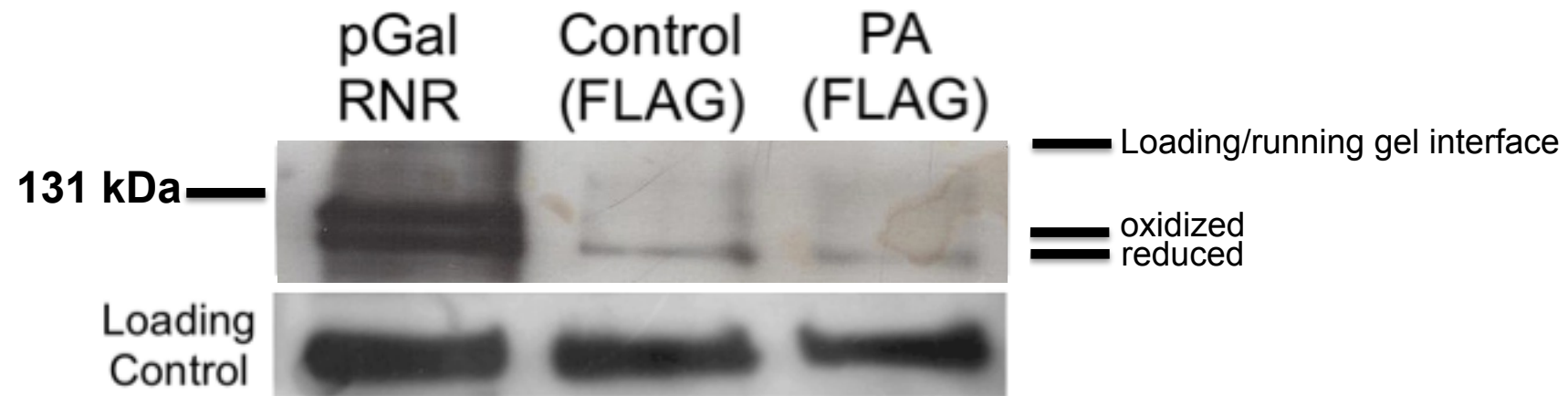

Figure S6

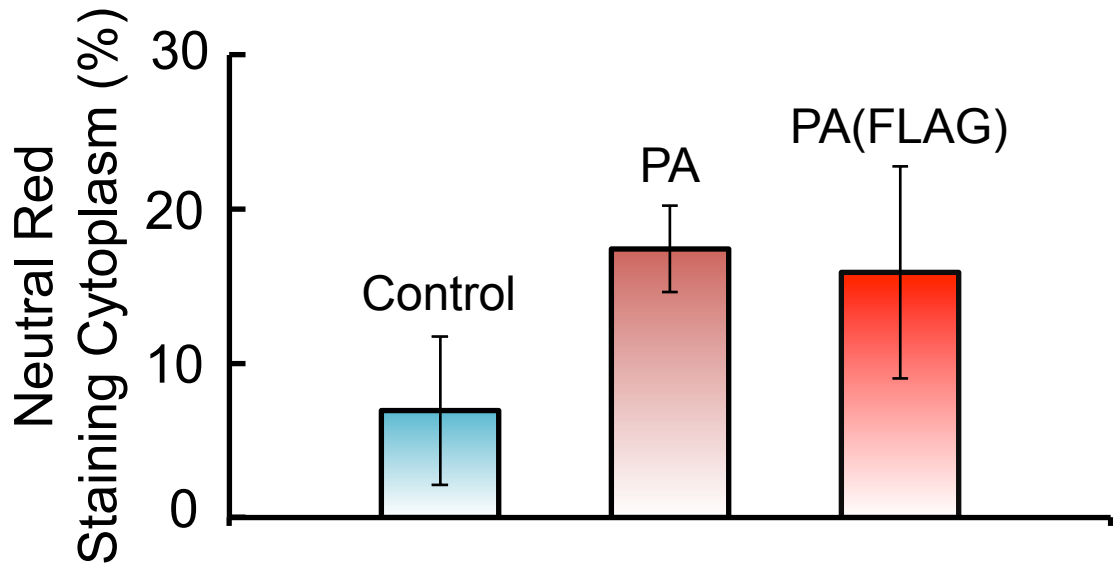

Figure S7

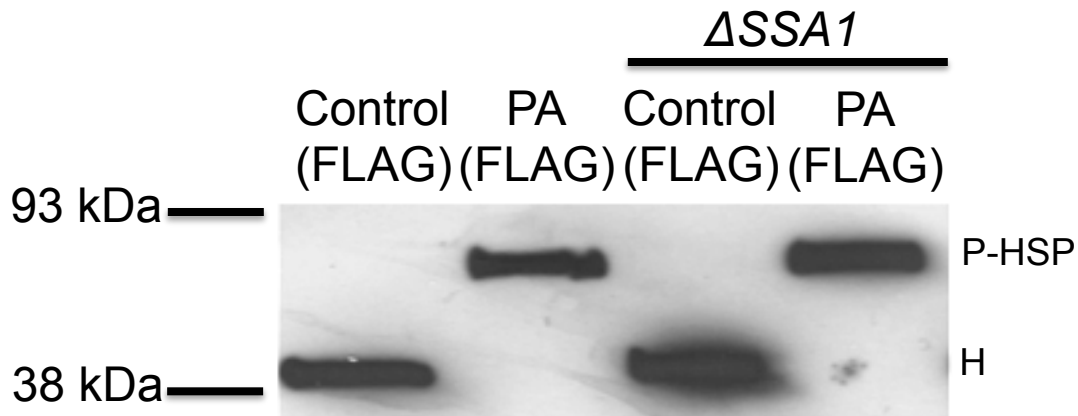

Supplement: Additional file 1: Figure S1 — In contrast to PA-expressing strains, yeast expressing the UN-24OR incompatibility domain have no discernable incompatibility-like phenotypes (P > 0.35). Yeast strain Y3068 is the untransformed parental strain, the “control” strain has hph integrated into the GAL1 locus, whereas hygunOR(788-929) and hygunOR(335-929) have OR incompatibility- domain replacements of GAL1. Cells were grown overnight at 30°C in YPD, washed in PBS, resuspended in YPD or YPRaf/Gal and grown with shaking until mid-log phase. Determination of MIC (A and B), granulated cytoplasm (C), and neutral red staining (D) were performed as described in the Methods section. Error bars indicate standard deviation from a minimum of 3 biological replicates for all panels. For both C and D a minimum of 100 cells were counted. Figure S2. Incompatibility-like phenotypes of control and PA strains were not significantly different when constructs were over-expressed by growing yeast in YPRaf/Gal (P > 0.05 in all cases). Briefly, cells were grown overnight at 30°C in YPD, washed in PBS, resuspended in YPRaf/Gal and incubated with shaking until mid-log phase. Cytoplasmic granulation (A), neutral red staining (B) and growth rate (C) analyses were performed as described in the Methods section. Error bars indicate standard deviation from 5 biological replicates. Figure S3. The frequency of dead cells tended to be greater in the strain over-expressing the PA construct than in the control strains, but did not significantly differ during lag, mid-log and stationary phase growth on YPD (P > 0.05 in all cases). Dead cells were recognized by deep blue color using the vital stain Evan’s Blue and light microscopy. OD600 was used to determine 2 growth phase based on the growth curve presented in Figure 3C. For vital staining, cultures were washed three times in PBS, resuspended in PBS, mixed with an equal volume of 1% w/v Evan’s Blue, held for 5 min at room temperature and examined at 40X using bright-field microscopy. A mini [file 1471-2180-13-63-S1.pdf]
