# Supplementary material for: Trans-species activity of a nonself recognition domain
Source: BMC Microbiol. 2013 Mar 22;13:63. doi: 10.1186/1471-2180-13-63 (PMC3618301; doi:10.1186/1471-2180-13-63)
Supplement: Additional file 2: Table S1 — Mascot results of anti-FLAG purified protein bands from hygFLAGunPA-expressing yeast grown in YPRaf/Gal. The ~54 kDa and ~85 kDa protein bands generated peptide sequences that corresponded to hygromycin phosphotransferase protein and Ssa1p, respectively. Table S2. Mascot results of anti-FLAG purified protein from yeast that lacked SSA1 and that expressed hygFLAGunPA. The ~ 85 kDa protein band yielded peptides that corresponded to the mitochondrial chaperone Hsp60 and to the cytosolic Hsp70 homolog, Ssb2p. Table S3. Yeast strains used in this study. [file 1471-2180-13-63-S2.pdf]

Supporting Tables

Table S1: Mascot results of anti-FLAG purified protein bands from hygFLAGunPA-expressing yeast grown in YPRaf/Gal. The ~54 kDa and ~85 kDa protein bands generated peptide sequences that corresponded to hygromycin phosphotransferase protein and Ssa1p, respectively.

Protein Candidate	Approx. MW on SDS-PAGE (kDa)	Theoretical MW (kDa) of Protein Candidate	Mascot Score	Peptide Sequences
Hygromycin phosphotransferase protein	54	38.32*	111	- AFSFDVGGR<br>- FDSVSDLMQLSEGEESR<br>- SAAVWTDGCVEVLADSGNR + Carb (C)
Heat Shock Protein 70 (Ssa1p)	85	69.615	325	- DAGTIAGLNVLR<br>- FEELCADLFR + Carb (C)<br>- SQVDEIVLVGGSTR<br>- TTPSFVAFTDTER<br>- ARFEELCADLFR<br>- NFTPEQISSMVLGK

* This is the theoretical MW of the hygromycin phosphotransferase protein without in-frame addition of the FLAG epitope and the *un-24*^{PA} incompatibility domain. The estimated MW of the hygFLAGunPAp monomer is 54 kDa.

Table S2: Mascot results of anti-FLAG purified protein from yeast that lacked *SSA1* and that expressed hygFLAGunPA. The ~ 85 kDa protein band yielded peptides that corresponded to the mitochondrial chaperone Hsp60 and to the cytosolic Hsp70 homolog, Ssb2p.

Protein Candidate	Approx. MW on SDS-PAGE (kDa)	Theoretical MW of Protein Candidate (kDa)	Mascot Score	Peptide Sequences
Heat shock protein Hsp60 precursor, mitochondrial	85	61	401	- VGGASEVEVGEK<br>- NVLIEQPFGPPK<br>- GSIDITTTNSYEK<br>- VLDEVVVDNFDQK<br>- GVETLAEAVAATLGPK<br>- TLEDELEVTEGMR<br>- NEAAGDGTTSATVLGR<br>- ISSIQDILPALEISNQSR
Heat Shock Protein 70 (Ssb2p)	85	66.5	333	-TGLDISDDAR<br>-STLEPVEQVLK<br>-FEDLNAALFK<br>-VTPSFVAFTPQER<br>-TFSPQEISAMVLTK<br>-AVITVPAYFNDAQR<br>-IINEPTAAAIAYGLGAGK<br>-VIDVDGNPVIEVQYLEETK<br>-LESYVASIEQTVTDPVLS SK

Table S3: Yeast strains used in this study.

Strain	Genotype	Reference
Y2454	*MATα mfa1Δ::MFA1pr-HIS3 can1Δ ura3Δ0 leu2Δ0 his3Δ1 lys2Δ0*	[1]
Y3068	*MATa can1Δ::MFA1pr-HIS3 ura3Δ0 leu2Δ0 his3Δ1 lys2Δ0*	[1]
YPL234CΔ	*MATa/A his3Δ1/his3Δ1 leu2Δ0 /leu2Δ0 lys2Δ0/LYS2 MET15/met15Δ0 ura3Δ0 /ura3Δ0 YPL234CΔ::G418^{R}*	[2]
YAL005CΔ	*MATa/A his3Δ1/his3Δ1 leu2Δ0 /leu2Δ0 lys2Δ0/LYS2 MET15/met15Δ0 ura3Δ0 /ura3Δ0 YAL005CΔ::G418^{R}*	[2]
p*GAL-RNR1*	*MATa ura3Δ0 leu2Δ0 his3Δ1 met15Δ0 pGAL-RNR1*	[3]

References

1. Tong AHY, Evangelista M, Parsons AB, Xu H, Bader GD, Page N, Robinson M, Raghibizadeh S, Hogue CWV, Bussey H *et al*: **Systematic genetic analysis with ordered arrays of yeast deletion mutants**. *Science* 2001, **294**(5550):2364-2368.
2. Giaever G, Chu AM, Ni L, Connelly C, Riles L, Veronneau S, Dow S, Lucau-Danila A, Anderson K, Andre B *et al*: **Functional profiling of the Saccharomyces cerevisiae genome**. *Nature* 2002, **418**(6896):387-391.
3. Sopko R, Huang D, Preston N, Chua G, Papp Bz, Kafadar K, Snyder M, Oliver SG, Cyert M, Hughes TR *et al*: **Mapping pathways and phenotypes by systematic gene overexpression**. *Molecular Cell* 2006, **21**(3):319-330.
